# Super-Enhancers Dysregulations in Hematological Malignancies

**DOI:** 10.3390/cells11020196

**Published:** 2022-01-07

**Authors:** Yannis Belloucif, Camille Lobry

**Affiliations:** INSERM U944, CNRS UMR7212, Institut de Recherche Saint Louis, Université de Paris, 75010 Paris, France; yannis.belloucif@inserm.fr

**Keywords:** enhancers, super-enhancers, regulatory elements, acute myeloid leukemia, hematological malignancies, enhancer hijacking, leukemia

## Abstract

Hematological malignancies affecting either the lymphoid or the myeloid lineages involve epigenetic mutations or dysregulation in the majority of cases. These epigenetic abnormalities can affect regulatory elements in the genome and, particularly, enhancers. Recently, large regulatory elements known as super-enhancers, initially identified for their critical roles in cell-type specific expression regulation of genes controlling cell identity, have been shown to also be involved in tumorigenesis in many cancer types and hematological malignancies via the regulation of numerous oncogenes, including MYC. In this review, we highlight the existing links between super-enhancers and hematological malignancies, with a particular focus on acute myeloid leukemia, a clonal hematopoietic neoplasm with dismal outcomes, resulting in an uncontrolled proliferation of myeloblasts, abnormally blocked during differentiation and accumulating within the patient’s bone marrow. We report recent works, performed during the last few years, treating this subject and consider the possibility of targeting oncogenic regulatory elements, as well as the effectiveness and limitations reported so far for such strategies.

## 1. Introduction

Hematological malignancies are blood disorders affecting either the myeloid or lymphoid lineages. Acute myeloid leukemia (AML) is an aggressive type of cancer affecting the myeloid lineage of white blood cells. Accumulation of mutations within a myeloid progenitor, the myeloblast, can lead to a block of differentiation, therefore, preventing its maturation. When combined with other mutations, allowing an uncontrolled proliferation of cells, this differentiation block can result in an accumulation of leukemic myeloblasts within the bone marrow, at the expense of normal hematopoietic cells. Eventually, blasts can accumulate in peripheral hematopoietic organs, such as the spleen and liver [1].

AML is considered a very diverse and heterogeneous disease, composed of a number of subtypes, in part due to the various cells of origin that accumulate mutations [2]. Several genetic alterations are responsible for the development of AML, which is known to be associated with clonal evolution, in particular during relapse. AML genomes, in general, carry several mutations, their number being related to patient’s age, and many frequently mutated genes have been reported across the years, such as *DNMT3A* [3], *FLT3* [4], *NPM1* [5], *IDH1* [6], *IDH2* [7], *WT1* [8], *EZH2* [9,10], *RUNX1* [11], *PTPRT* [12], *PHF6* [13], *ETV6* [14], *ASXL1*, *MLL*, *CEBPA*, *KIT* [15], *TET2* [16], *KRAS* [17], *WAC*, *SMC3*, *DIS3*, *DDX41*, and *DAXX* [18].

In addition to point mutations, chromosomal translocations are commonly found in AML, particularly in pediatric cases and result in so-called oncofusion proteins. These chimeric proteins often involve transcription factors, with, for instance, one that allows the fusion protein to retain the DNA binding motif of the wild type protein, and another offering a domain permitting interaction with a corepressor complex [19]. These kinds of oncofusion proteins can impair the expression of genes implicated in myeloid differentiation, through different mechanisms, contributing to leukemic transformation. AML1-ETO and PML-RARa fusions are well-known examples, which were demonstrated to regulate the expression of many of the genes involved, notably in differentiation, cell survival, DNA repair, and signal transduction [20]. In hematological malignancies, and in particular in AML, mutations directly affecting genes involved in epigenetic modifications or other alterations impacting the epigenome have been identified in over 70% patients [21]. These abnormalities can reshape the global epigenetic landscape and affect the key regulatory elements responsible for the control of gene transcription, such as enhancers or insulators. In this review, we focus on one particular recently identified type of regulatory element, termed super-enhancers (SE). We review recent work and advances pinpointing their crucial roles and mechanisms of action in gene regulation, pertaining to hematological malignancies. We, furthermore, develop some examples of the SE dysregulations involved in hematological malignancies, with a particular focus on AML, and discuss the pertinence and effectiveness of targeting oncogenic SE.

## 2. Enhancers and Super-Enhancers

### 2.1. Characteristics

Genomes are composed of coding and non-coding genes, as well as regulatory elements such as insulators and enhancers. It was estimated by the ENCODE project that the human genome is composed of around 400,000 putative enhancers, completely outnumbering the 20–25 thousand protein-coding genes they regulate [22]. Enhancers are defined as cis-acting DNA sequences capable of increasing the transcription of one or several genes. Those regulatory elements are composed of clusters of transcription factor (TF) binding sites, each of which is responsible for the activation or repression of transcription. Enhancers can be found upstream or downstream of their target genes, or even inside or overlapping the gene body of a gene [23]. In general, enhancers are able to regulate gene expression independently of their orientations, at various distances from their target promoters. In metazoans, this distance fluctuates between 100 bp and several kilobases or megabases within the same chromosome, as was discovered with shadow enhancers [24]. Importantly, enhancers are the basis of a fundamental characteristic of cellular identity, which is the existence of the differential expression of genes across cell-types and developmental stages. Indeed, those cis-regulatory regions are known for their essential role in providing transcriptional tissue-specificity, as well as developmental-specificity.

Recently, another type of regulatory elements has been discovered and is currently being studied, particularly for its involvement in several types of cancers, namely super-enhancers (SE). SE are defined as large genomic regions, corresponding to clusters of enhancer elements, spanning on average more than 20 kb [25,26]. They are characterized by several main features. First, by high levels of active enhancer histone tail modifications, such as H3K27ac and H3K4me1. Second, by an important binding enrichment of transcriptional co-activators and enhancer-associated factors, such as the mediator complex (more particularly MED1), bromodomain-containing proteins like BRD4, as well as an increased binding of p300 [27].

**Figure 1:**
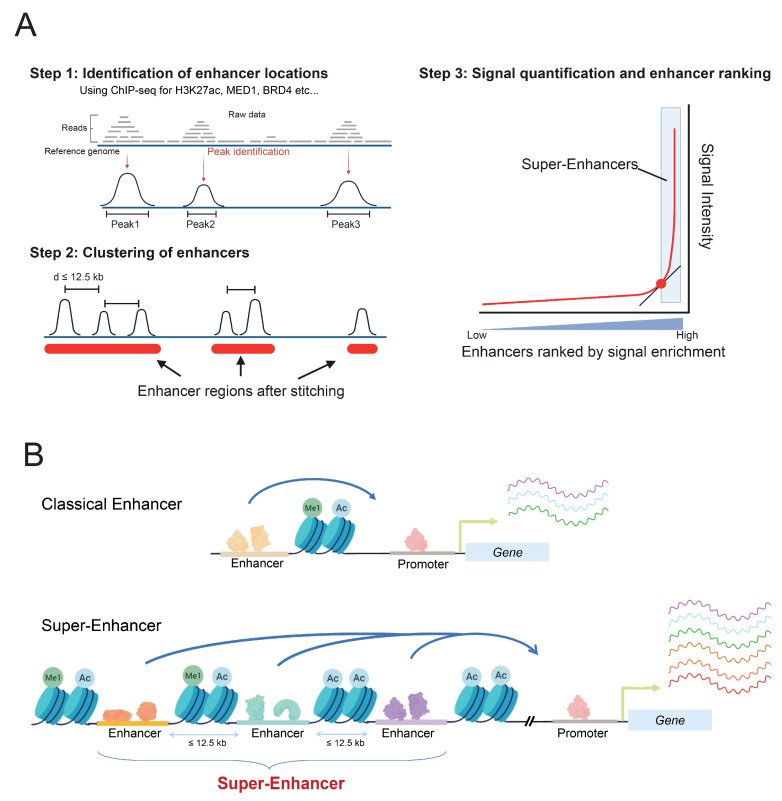
Definition of super-enhancers. (**A**) Steps of identification and ranking of super-enhancers: reads from ChIP-seq for enhancer specific factors (such as MED1, BRD4) or histone modifications (H3K27ac, H3K4me1) are mapped to the reference genome and specific enrichment peaks are determined bioinformatically, then peaks closer than 12.5 kb are stitched together and the overall read signal intensity within these stitched areas is counted. Stitched regions are finally ranked by signal intensity, and super-enhancers are defined as regions whose intensity is above the tangent of slop 1. (**B**) Super-enhancers, as clusters of enhancers, are larger, present a higher TF and RNA pol II binding density, and induce a higher gene transcription compared to regular enhancers.

SE were defined in silico by stitching together closely distributed enhancers, identified by their H3K27ac or MED1 enrichment, and separated from one another by a distance excluding randomness, on average less than 12.5 kb [26] (Figure 1A). Those SE differ from regular enhancers by their size, their TF binding density, and their higher potential to activate the gene expression of adjacent genes [26,28], compared to classical enhancers (Figure 1B). Moreover, RNA pol II is found at greater levels on SE, thereby resulting in higher levels of eRNAs production, referred to as super-enhancer RNAs (seRNAs) [29].

SE are often found near lineage-specifying genes, driving their expression and, thereby, controlling cell identity. In addition, for a great number of SE, their activation states are cell-type specific, making them key regulators of cell-type differentiation [25]. Some of them are implicated in various important processes, including pluripotency in murine embryonic stem cells, regulating the expression of *OCT4*, *NANOG*, and *SOX2* genes [26]. Furthermore, several SE are located near well-known oncogenes, such as *MYC* [30], *ERG* [31,32], *KIT* [33], *BCL2* [34], or *TAL1* [35], suggesting that they might also be key players in the pathogenesis of cancers.

### 2.2. Modes of Action in Gene Regulation

Regarding the mode of action of active enhancers in gene expression regulation, it relies on the three-dimensional organization of the chromatin, more precisely through the existence of topologically-associating domains (TADs) and chromatin loops. TADs are small chromosomal domains defined by the presence of frequent long-range interactions between loci located in the same domain, and less frequent contacts between loci of different domains. Those genomic regions have specific sizes and are delimited by boundaries, often containing CTCF-binding sites, housekeeping genes, and short interspersed elements [36,37]. Within TADs, chromatin loops are at the center of these enhancer–promoter interactions, allowing direct and specific communication between these two regulatory elements mediating gene expression regulation [38,39].

**Figure 2:**
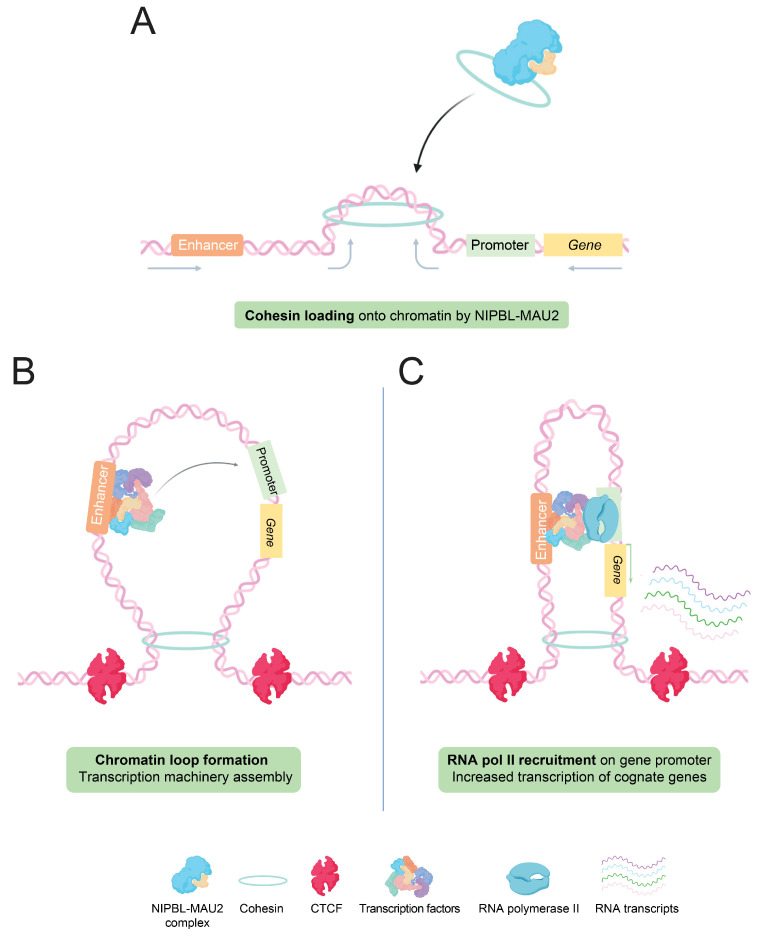
Loop extrusion model: Enhancer-promoter interactions through chromatin loops formation. (**A**) Cohesin rings are loaded onto chromatin by the NIPBL-MAU2 protein complex, at the base of the future loop. Some papers suggest that chromatin is actively extruded through the loop by the action of SMC cohesins. (**B**) The encounter of cohesin and CTCF proteins on each side of the loop is thought to block the progression of the loop extrusion. Another hypothesis further proposes that transcription factors, accumulating within the enhancer, could then be ‘exchanged’ or transferred between enhancer and promoter, through the proximity allowed by the loop. Such interactions could facilitate the recruitment of the transcriptional machinery, as well as RNA pol II, at the target gene promoter. (**C**) The increased concentration of transcription factors at gene promoters permitted by enhancer and RNA pol II recruitment ultimately results in a stronger transcription of cognate genes.

TADs and chromatin loops depend on cohesins, ATPase proteins member of the ‘structural maintenance of chromosomes’ (SMC) family, characterized by a ring shape, able to surround DNA strands [40]. The mechanism by which loops and TADs are formed in vivo is still not fully delineated, but several hypotheses have been proposed. The main hypothesis is the presence of extrusion loops, produced through an active extrusion of DNA by cohesins (Figure 2A), until the encounter of a ‘road-block’ such as the CTCF protein, interacting with specific DNA sequences, and impairing physically the extrusion [41,42] (Figure 2B). In that model, cohesins are localized at the loop base and their extrusion capacity depends on their ATPase activity [42,43]. This model was recently strengthened by in vitro studies, which showed that cohesin complexes and NIPBL-MAU2, a protein complex allowing the loading of cohesin onto chromatin, are both required for this cohesin-mediated loop extrusion [44].

With these notions, the looping model suggests that the transcription machinery, loaded at first on active enhancers, could then be ‘transposed’ through a potential exchange mechanism to its assigned promoter, facilitated by the physical proximity accorded by the loop [45]. Through this function, enhancers increase the amount of active transcriptional machinery on specific promoters, thereby resulting in higher transcription of underlying genes (Figure 2C).

Similarly to what was shown with regular enhancers, SE also rely on chromatin loop formation, to be brought closer to their target genes. ChIP-seq data analysis of CTCF and cohesin component binding showed an important enrichment within SE regions, suggesting the formation of extrusion loops mediated by cohesin complexes and CTCF [46]. However, whether SE act as a whole to regulate gene expression, or each of the enhancers composing the SE have an individual effect on their associated target gene(s), remains to be clearly demonstrated. In the former scenario, SE function as one regulatory element, able to increase as a whole the transcription of genes localized in the same chromatin loop. One can envision a possible synergy between enhancers, where each of the enhancers composing the larger regulatory element might have a required effect, in order for that SE to function. In that scheme, we can hypothesize that inhibiting one of the enhancers composing that SE would disrupt the entire regulatory element, hence massively impacting the expression of the associated gene(s). An example supporting this mechanism was reported in 2016 by H. Youn Shin and colleagues, where they studied the *Wap* super-enhancer, composed of three enhancers, in mice carrying mutations within Stat5 binding sites [47]. They demonstrate that the deletion of Stat5 binding sites within the most proximal enhancer resulted in the abrogation of the entire SE, hence almost completely losing the expression of *Wap*. The two other enhancers seemed to have a less important roles in its transcription, therefore adding a notion of enhancer-hierarchy within a SE region [47].

A second hypothesis, introducing the notion of additivity, would be that a SE acts through the added and independent functions of all or part of its attributed enhancers, thereby explaining the higher transcription obtained compared to a regular enhancer. This mechanism would be supported if the inhibition of one of the enhancers resulted in a mild/non-significant decreased transcription of the cognate gene(s) [48]. A recent study published in 2018 focused on a SE regulating *MYC* expression in hematopoiesis. The authors developed mice carrying deletions of individual enhancer modules composing that SE and showed that these deletions induced milder effects on *MYC* expression, which were cell-type specific, with some modules affecting *MYC* expression in HSCs, others in B-cells. Their results, therefore, suggest that *MYC* expression through this SE is due to a combination of independently acting enhancers, allowing a cell-type specific regulation of this oncogene, strongly supporting the additive mechanism [30]. Another strong example is the in vivo dissection of the ⍺-globin SE in murine erythroid cells. The authors individually deleted five of the regulatory elements composing this SE using homologous recombination; hence, generating several mouse models. Interestingly, they found that none of the five enhancers were significantly required to control the expression of any of the globin genes, strongly supporting the additive hypothesis [49].

## 3. Enhancers and Super-Enhancers in Hematological Malignancies: Enhancerophathies

An increasing number of publications have highlighted the relationship between SE and diseases. Interestingly, by looking at the distribution of single nucleotide polymorphism (SNPs) associated with diseases of 86 different cell-types, Hnisz and colleagues highlighted that 64% of the SNPs occurring in non-coding regions were actually found within enhancers and were significantly more enriched in SE [25]. This indicates the crucial role of mutations within super-enhancers, linked to several genetic diseases such as Alzheimer’s disease, with 19% of related SNPs occurring in SE, type 1 diabetes (19%), systemic lupus erythematosus (33%), and cancer [25]. The link between SE and cancer emerged soon after the discovery of these regulatory elements, in 2013. By identifying SE and their associated genes in 18 cancer cell lines using H3K27ac ChIP-seq data, the same authors observed that many well-known oncogene drivers were associated with SE. By further comparing those cancer cells with related normal cells, they were able to show that some of those SE were not active in normal cells, thereby suggesting that some SE may be aberrantly activated in cancer cells, particularly near oncogenes, during tumorigenesis. An example of this observation is given by the *MYC* oncogene, where SE were found near the *MYC* gene locus in multiple cancer cell-types, but not in their related normal cells. Several mechanisms can explain the acquisition of de novo SE in cancer cells [25]. These include insertion/deletions (indels) of DNA sequences containing transcription factor binding sites, DNA translocations involving cis-regulatory elements, transcription factor overexpression that could hijack and activate enhancer regions, SE focal amplification (copy number gain), and potentially other unknown mechanisms. Hematological malignancies, and particularly leukemias, including acute myeloid leukemia, show a high frequency of genetic lesions, impacting epigenetic regulations and, therefore, enhancer and SE activation. These different mechanisms will be highlighted and discussed using examples in hematological malignancies and, particularly, AML.

### 3.1. Point Mutations and Indels

**Figure 3:**
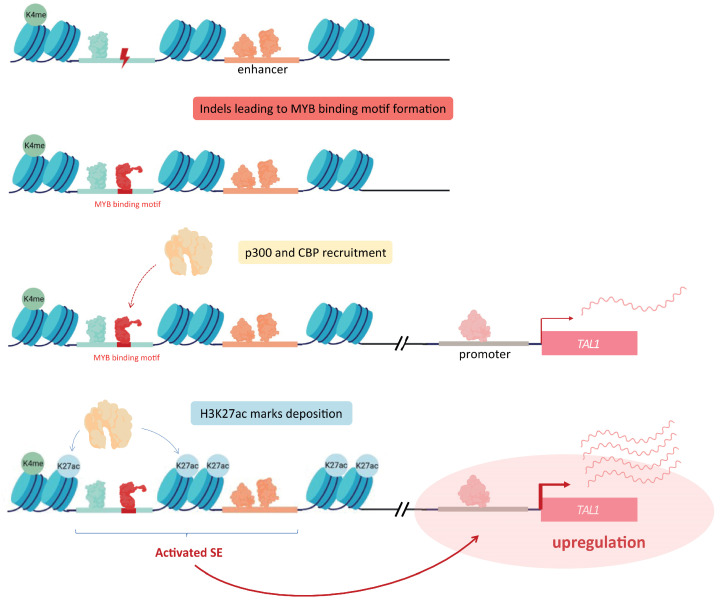
Indels inducing the introduction of MYB binding sites near TAL1. Somatic mutations acquired within a SE located near the *TAL1* oncogene allow the production of MYB binding sites. Accumulation of MYB at these sites is responsible for the recruitment of p300 and CBP, leading to the deposition of H3K27ac marks and SE activation. Altogether, this results in the overexpression of the associated gene, here being *TAL1*.

Enhancer regions can be subject to several kinds of mutations, affecting either one or several bases. Such modifications can lead to the aberrant production of SE, through the formation of additional TF binding sites. As an example, Mansour and colleagues further developed the notion of oncogenic SE, by identifying heterozygous somatic mutations creating a de novo binding motifs for the master transcription factor MYB in a non-coding region proximal to the *TAL1* gene in a subset of T cell acute lymphoblastic leukemia (T-ALL). This enables an abnormal binding of MYB within these newly created sites, recruiting other factors, such as CBP and p300, and ultimately resulting in the formation of a SE upstream of the *TAL1* oncogene, inducing its overexpression [35] (Figure 3).

A few years later, a comprehensive effort led by the teams of M. R. Mansour, T. A. Look, and R. A. Young demonstrated, through a combination of ChIP-seq and DNA sequence alignment, that some somatic insertions within the non-coding genome are involved in the formation of active enhancer regions in a broad panel of 102 cancer genomes. They, in particular, validated an insertion creating a novel enhancer driving the *LMO2* oncogene expression in T-ALL [50]. Although this study encompassed a couple of AML cell lines showing insertions of enhancer elements, further large-scale studies are required to extend and validate such findings in AML.

Similarly in T-ALL, R. A. Young’s team showed that the recurrent microdeletions found in tumor cells genomes were responsible for the suppression of CTCF sites, normally mediating the boundaries of chromatin loops that contain enhancers/SE and promoters of their associated genes. Using ChIA-PET, in order to map insulated neighborhoods and chromatin-interactions mediated by cohesin, they identified that such micro-deletions of CTCF binding sites lead to the disruption of initial loop boundaries; hence, resulting in the aberrant activation of proto-oncogenes such as *TAL1* and *LMO2* involved in this disease [51].

Another group established, in gastrointestinal stromal tumors (GISTs), that a global hyper methylation of the genome greatly affects specific CTCF binding sites in SDH (succinate dehydrogenase) deficient GISTs cells. This hypermethylation of CTCF sites is responsible of topological rearrangements activating oncogene programs, notably involving *FGF3/4* and *KIT* [52]. This has not yet been shown in any hematological malignancies.

Mutations affecting cohesin complex genes are highly reported in Down-syndrome associated acute megakaryoblastic leukemia (DS-AMKL), representing more than 50% of patients. Such mutations are thought to alter gene expression through a chromatin accessibility rewiring, as well as epigenetic complex targeting. These mainly affect genes encoding cohesin complex subunits corresponding to *SMC3*, *STAG2*, *RAD21*, and *SMC1A* [21]. On the other hand, mutations either affecting or deleting the insulator binding protein CTCF are also frequently found in DS and non-DS-AMKL patients (respectively, 20% and 21%) [53].

Both, hyper-methylation of CTCF sites and mutations of cohesin complex factors can lead to a loss of CTCF binding at TAD boundaries, potentially enlarging the chromatin loops. This enlargement can result in the interaction of oncogene promoters with normally unrelated enhancer structures, and, hence, alter their regulation.

### 3.2. Focal Amplification of Super-Enhancers

Focal amplifications including copy-number variations (CNV) are important players in cancer development. Focal amplifications of SE can be detected in many types of cancer. Zhang and colleagues combined a somatic copy number analysis of 12 cancer cell-types and a tissue-specific epigenetic profiling to identify SE regions presenting a copy number gain. This allowed them to show that several SE regions presenting copy number gains were associated with the overexpression of four neighboring cancer-associated genes, involving again the *MYC* oncogene [54]. Such examples are very well documented for solid cancers, but less so in hematological malignancies.

In T-ALL, a *MYC* enhancer, notably controlled by NOTCH1, was shown to be frequently targeted by chromosomal duplication. This regulatory element is located within a SE region upstream of the *MYC* promoter, and is able to interact directly with it to induce *MYC* expression, which in turn has an important role in thymocyte development and NOTCH1-induced T-ALL [55].

Another study performed in AML cells showed that another *MYC* SE located 1.7 Mb downstream of its transcription start site (TSS) corresponds to a frequently focally-amplified region in approximately 3% of AMLs. They also demonstrated that this region indeed directly interacts with *MYC* promoter through chromatin looping, notably mediated by a SWI/SNIF component named BRG1, which results in *MYC* expression maintenance and leukemic cell proliferation [56].

Other studies performed on solid malignancies demonstrated that such SE amplifications are able to upregulate associated oncogenes in a lineage-specific way, henceforward promoting tumorigenesis. Altogether, this suggests that SE focal amplification is a common mechanism, leading to the upregulation of driver oncogenes in several cancer types.

### 3.3. Translocations Involving Super-Enhancers

Chromosomal translocations are very frequent events involved in hematological malignancies and leukemogenesis, notably through the production of oncofusion proteins. However, stochastic chromosomal rearrangements without gene fusions are also important mechanisms, allowing the reconciliation of a given oncogene and an enhancer or SE, resulting in its abnormal overexpression. Several examples of such phenomenon were reported in different hematological malignancies.

In blastic plasmacytoid dendritic cell neoplasm (BPDCN), another subtype of acute leukemia, it was reported that a translocation specifically found in plasmacytoid dendritic cells allowed the association of the *RUNX2* SE with *MYC* promoter, thereby permitting their concomitant expression, involved in the disease [57]^.^

This is the case in AML, with the inv(3)/t(3;3) that is known to be associated with an increased expression of the *EVI1* gene, encoding a stem cell regulator. In this example, Ruud Delwel’s team showed that a distal enhancer regulating *GATA2* expression is relocated upstream of *EVI1* promoter, allowing an ectopic expression of the latter gene and, furthermore, demonstrating a concomitant *GATA2* haploinsufficiency in this AML model [58].

In multiple myeloma (MM), the *MYC* oncogene is frequently involved in chromosomal translocations [59], often repositioning it near genes associated with SE, such as immunoglobulin genes (e.g., *IgH*, *IgK*, *IgL*), *FOXO3*, *PRDM1*, and others, leading to its overexpression. In addition, fusions between *MYC* and *IgH* or *IgL* loci were found in 15% of treated and untreated MM tumor cells [60]. Similarly, chromosomal translocations juxtaposing SE to the *MYB* locus were reported in adenoid cystic carcinoma, with 3-C (chromosome conformation capture) data supporting a direct interaction between translocated SE and *MYB* promoter, allowing an increased transcription of this oncogenic TF. However, surprisingly in this case, *MYB* is also able to bind to the newly positioned SE, resulting in a positive feedback loop, sustaining its aberrant expression [61].

### 3.4. Super-Enhancer Hijacking

Enhancer and SE hijacking refers to a mechanism by which an abnormally overexpressed TF binds to an inactive or poised enhancer already located near a given oncogene, recruiting other factors and chromatin remodelers. This binding allows the aberrant activation of the considered enhancer/SE and, thereby, upregulates its associated oncogene. This is the case with several hematological malignancies, including B-cell acute lymphoblastic leukemia (B-ALL), where it was shown that STAT5 highly binds SE, notably regulating *MYC* and *BCL2L1*, thought to be a defining feature of B-ALL, by inducing B-cell transformation [62].

More recently, Ruud Delwel’s team also showed, in AML cells harboring the t(3;8)(q26;q24) translocation, that *EVI1* oncogene is able to hijack *MYC*’s SE. This mutation also leads to the overexpression of *EVI1* through a facilitated enhancer–promoter interaction, allowed by the multiple CTCF binding sites contained within this *MYC*-SE [63].

Additionally, studies in AML highlighted the function of the BRG1 ATPase, part of the SWI/SNF chromatin remodeling complex, whose interactions with *MYC* SE are notably involved in chromatin loop maintenance, allowing SE-*MYC* promoter interactions, in this way promoting *MYC* expression in leukemic cells [56].

**Figure 4:**
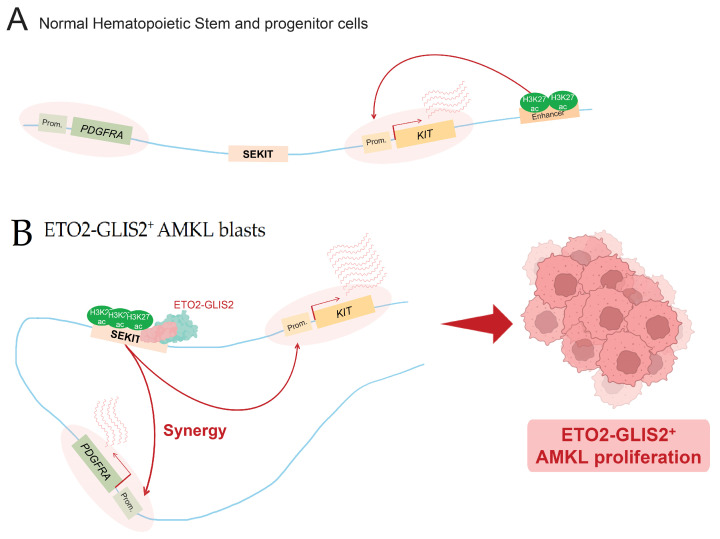
Super-enhancer hijacking by ETO2-GLIS2 fusion. (**A**) In normal hematopoietic stem and progenitor cells, *PDGFRA* gene is not expressed and *KIT* expression is mainly driven by an enhancer located 3′ of its transcription termination site. (**B**) In AMKL blasts, ETO2-GLIS2 fusion is able to bind and hijack a de novo super-enhancer, termed SEKIT. This leads to the abnormal activation of SEKIT and the subsequent expression of two tyrosine kinase encoding genes, *KIT* and *PDGFRA*, involved in ETO2-GLIS2^+^ AMKL proliferation.

Another important example concerns AMKL cells expressing the ETO2-GLIS2 oncofusion protein. ETO2-GLIS2 has been shown to directly bind DNA at SE regions and to be involved in their activation, as showed by Thirant and colleagues [32]. Activated SE are then able to upregulate their associated genes, which is notably the case for the *ERG* oncogene, playing an important role in the maintenance of this specific subtype of AML [32]. This fusion protein was shown to bind many SE specifically identified in AMKL patient cells, notably a SE named SEKIT, specifically bound by the fusion protein and activated in AMKL cells expressing ETO2-GLIS2. Benbarche and colleagues showed that in normal hematopoietic cells, as well as other AML subtypes, this particular region is inactive and *KIT* expression is driven by other enhancers, such as a previously identified classical 3′ enhancer [33] (Figure 4A). In ETO2-GLIS2+ AMKL cells, they showed that fusion induces the activation of a de novo super-enhancer, which allows the upregulation of its associated oncogenes, *KIT* and *PDGFRA*, essential for leukemic cell proliferation [64] (Figure 4B). AML1-ETO can also be mentioned in this Section 3.4, because of its ability to transactivate the expression of *KIT* in AML cells, by notably mediating extrusion loops, allowing the interactions of *KIT* promoter and one of its enhancers [65].

A final example of such enhancer-hijacking is provided by AML with the GATA2 SE translocated near *EVI1*′s promoter. By using CRISPR-Cas9, the authors demonstrated that a single enhancer contained within this GATA2 SE is composed of MYB binding sites, strongly required for *EVI1* overexpression in AML cells. In addition, the mutation of this MYB binding site within this specific SE leads to myeloid differentiation, as well as cell death [66]. However, interestingly, several examples of an opposite mechanism were recently reported, where some mutations affecting tumor suppressor genes abrogate the function of enhancers. Tultrup and colleagues showed in AML, that mutations affecting the *TET2* encoding gene, normally promoting DNA demethylation, are associated with hypermethylation of some enhancer regions [67]. The authors further demonstrated that the active enhancers targeted by this hypermethylation are specifically found in HSC, some of them being involved in myeloid leukocyte function, as well as immune response [67]. Kristian Helin’s team had already found that TET2 is predominantly recruited at open-chromatin regions that include enhancers. Here, they demonstrated that a majority of open-chromatin regions were less accessible through *TET2* loss [68]. Such DNA hypermethylation was reported to affect up to 25% of active enhancers within preleukemic hematopoietic cells mutated for *TET2* [69]. Such observations were also made with AML patients presenting *TET2* mutations, in which hypermethylated enhancers led to significant down-regulations of tumor suppressor genes, hence being involved in leukemogenesis [69].

### 3.5. Viral Oncogenes Activation of SE

TF overexpression is a frequent phenomenon in leukemia, and can be responsible for oncogenic SE activation. As mentioned, *TAL1* was found to be overexpressed in many T-ALL patient cells, resulting in the formation of an abnormal SE nearby the *MYC* locus [25]. However, such TF upregulation can also be obtained through viral infections. Indeed, it was reported that during an Epstein–Barr virus (EBV) infection on human B-cells, some TF encoded by EBV, as well as some host cell TF activated by this virus, are upregulated, leading to the formation of viral oncogene-mediated SE, particularly near pro-survival and antiapoptotic oncogenes, such as *RUNX3*, *MYC*, and *BCL2* [34]. Similarly, a study on cancer cells associated with papillomavirus (HPV) infections revealed that tandemly-integrated copies of the HPV16 genome were responsible for the formation of SE-like structures, and highly enriched in the binding of SE markers, such as BRD4, MED1, and H3K27ac histone marks. This SE-like element is in charge of the increased expression of the E6 and E7 viral oncogenes, resulting in an abnormal proliferation of those infected cancer cells [70]. To date, no example of SE activation by viruses has been reported in AML.

## 4. Targeting Oncogenic Super-Enhancers in Cancer Therapy: An Effective Approach?

Oncogenic SE, acquired during tumorigenesis by many types of cancer cells, induce the upregulation of oncogenes and can, as a result, induce a phenomenon known as oncogene addiction. This concept was introduced by Weinstein and Joe in 2006, to explain how cancer cells can become highly dependent on one or several oncogenes, some of them responsible for the dysregulation of transcriptional programs, allowing their increased proliferation and survival. They, therefore, postulated that repressing these key oncogenes represents an opportunity to inhibit cancer cell growth [71]. Additionally, it was already considered that enhancers with a high amount of TF binding sites (today, corresponding to SE) can have a higher sensitivity to subtle changes in TF concentration compared to classical enhancers. It is thought that these small changes can induce significant effects on the expression of associated genes [72].

The identification of oncogenic SE a decade later contributed to the idea that their specific inhibition could constitute interesting therapeutic options against cancer, since some of them were already demonstrated to regulate genes responsible for cancer cell addiction [73]. Moreover, targeting oncogenic SE could be one of the keys to selectively killing cancer cells, by affecting oncogenic transcription in a specific manner.

Several super-enhancer inhibitors have been developed, such as bromodomain and extra-terminal domain protein inhibitors (BETi), and cyclin-dependent kinase inhibitors (CDKi), both capable of inducing cancer cell death, notably, by inhibiting oncogenic SE transcription.

BRD4, a member of the BET family protein, is able to bind acetylated lysines of histones within TSS and SE, as well as acetylated lysines of TF. Thus, BRD4, through these interactions, contributes to the recruitment of TF at SE and mediates long range transcription activation [74].

Johannes Zuber and colleagues were the first to identify *BRD4* as an interesting therapeutic target in AML, by performing an RNAi screen targeting known chromatin modifiers. Indeed, they showed that the shRNA-mediated suppression of BRD4 led to in vitro and in vivo anti-leukemic effects [75].

A BRD4 inhibitor known as JQ1 was then studied in MM cells by the teams of Bradner and Mitsiades, showing in particular that its inhibition affected *MYC* expression and subsequent *MYC* target genes [76].

Two years later, Richard Young’s group revealed that JQ1 treatment led to a preferential depletion of BRD4 at SE. Such BRD4 loss was shown to be responsible for impaired transcription elongation at SE-associated genes, notably *MYC* [72]. JQ1′s effect on *MYC* has been confirmed in many other cancer types, including AML [75] and Burkitt’s lymphoma [77]. This is consistent with other results showing that BRD4 inhibition impedes the interaction between SE and their cognate gene promoters, associated with cell-specific repression of oncogenes and cell-death [78]. In MM, the inhibition of BRD4 by using the JQ1 inhibitor led to a preferential loss of BRD4, mediator, and P-TEFb (positive-transcription elongation factor) particularly at SE. This BRD4 inhibition was responsible for a general decreased transcription of genes associated to SE, which include the *MYC* oncogene [72].

On the other hand, CDK7, a subunit of TFIIH, phosphorylates, with other CDK proteins, the carboxy-terminal domain (CTD) of RNA pol II, promoting transcription initiation and elongation. THZ1, a CDK7 inhibitor, is able to prevent RNA pol II phosphorylation [79] and was demonstrated to lead to an accumulation of RNA polymerases at gene bodies [80], as well as a downregulation of SE-associated genes, including *MYCN* in neuroblastoma (NB) cells [81] and *RUNX1* in T-ALL [82], inhibiting, in this way, tumor growth.

Other inhibitors co-targeting the casein kinase 1 alpha (CK1⍺, known to suppress the activity of TP53 [83]), CDK7 and CDK9, were developed in Yinon Ben-Neriah’s lab. Combined inhibition of CK1⍺, CDK7, and CDK9 resulted in a stabilization of p53 and β-catenin production, downregulation of Mdm2 and disrupted many SE responsible for the expression of several important oncogenes that included *MYC*, *MYB*, and *MCL1* in MLL-AF9 induced and *Tet2^-/-^Flt3^ITD^* AML mice models [84].

The extensive use of BETi, such as JQ1, can lead to an acquired BETi resistance, mainly mediated by epigenetic remodeling and re-expression of BRD4 target genes [85]. Therefore, in order to find a weakness in BETi-resistant AML cells, some researchers tested a combined BETi with CDK7 inhibition treatment, and showed that targeting both BRD4 and CDK7 resulted in an interesting synergistic effect in vitro and in vivo, affecting BETi-resistant cell growth [86].

In addition, recently, S. Greg Call and colleagues showed that the nuclear receptor encoding gene NR4A1, known as a tumor suppressor in AML, as well as a drug inducing its expression, named Dihydroergotamine, are able to inactivate an oncogenic SE associated with *MYC* expression. The mechanism involves a loss of important coactivators at the SE, including BRD4, Mediator, and p300; hence, decreasing H3K27ac marks [87].

## 5. Limitations of Super-Enhancer Targeting

These SE inhibitors are promising, with much data supporting their ability to efficiently kill different types of cancer cells, notably, by targeting oncogenic regulatory elements. However, questions remain regarding the specificity of those inhibitors and their effects on normal cells. By considering the results from Chipumuro and colleagues testing the efficacy of THZ1 with increasing concentrations on *MYCN*-amplified NB cells and untransformed cells (murine fibroblasts), 100 nM of THZ1 is sufficient to massively decrease the viability of *MYCN*-amplified NB cells, to 20% or less considering cell lines, but also leads to a decreased viability of untransformed cells, reaching 50% [81]. Studies about JQ1 also reported a related toxicity in neuronal derivatives, inducing their decreased proliferation and viability [88], as well as the downregulation of genes involved in self-renewal, cell cycle, DNA replication, and mitosis in mesenchymal stem cells [89]. We can also mention other studies that showed that a JQ1 treatment on male mice resulted in abnormal fertility, more precisely decreasing seminiferous tubule area, size of testis, and the number of spermatozoa, as well as their motility [90]. This demonstrates that those inhibitors, through their unspecific modes of action, also have the potential to affect ‘normal’ SE and harm untransformed cells.

Several clinical trials using BETi structurally similar to JQ1 have been performed over the years. OTX015, a molecule able to bind to BRD2/3/4 and prevent them from binding acetylated histone H4, was tested in a dose-escalation study on 36 AML patients [91].

Therefore, finding a way to direct such inhibitors, specifically at cancer cells or specifically at oncogenic SE, could lead to an improvement in SE research and cancer therapy, enabling us to selectively repress key transcriptional regulators involved in tumorigenesis or cancer state maintenance, already shown to effectively decrease tumor cell viability and growth [92]. In addition, this requires overcoming an upstream challenge, being able to discriminate oncogenic SE from normal SE. As an example, the BENC super-enhancer was suggested as a therapeutic target for *MYC*-addicted AML [30,54,72].

## 6. Concluding Remarks

The main objective of this review was to provide general information about super-enhancers and their involvement in several hematological malignancies, with a particular focus on AML.

We discussed the interest in considering SE in the context of cancer, first as potential novel therapeutic targets with the development of inhibitors, such as JQ1 or THZ1, but more importantly as nodules at the basis of gene networks, whose study can allow the identification of numerous aberrantly expressed gene networks, including potential oncogene addictions, involved in the development and progression of leukemia.

Most of the oncogenic SE discovered so far were implicated in chromosomal translocations or indels, and were, therefore, identifiable through DNA sequencing of cancer cells [35,57]. Here, we mentioned that some of the mechanisms responsible for de novo oncogenic SE formation do not directly involve the SE itself, and instead lean on the overexpression of some TF that will result in oncogenic SE activation. Such cancer-restricted regulatory elements, produced from these kinds of processes, might be more laborious to find through DNA sequence analysis alone, considering that some of the mechanisms might still be unknown.

Chapuy et al. previously suggested, in 2013, the idea that studying SE could help in the identification of cancer dependencies [73]. Through this approach, it seems possible to discover oncogenes responsible for dependency in some cancer cells. It is known that cancer cells do not depend on every oncogene, some of them being necessary for their survival, others for their uncontrolled proliferation. Hence, some might not be, *per se*, mandatory for the viability of malignant cells, but can give them an additional fitness. This could lead to the development of novel repressors/inhibitors, targeting some of those addictive genes, either at the gene or at the protein level, as well as through combined chemotherapy development. The combination of chemotherapeutic agents brings many advantages, especially dosage lowering, which contributes to decreased treatment toxicity and offers a better specificity, by mainly affecting cancer cells.

We discussed the emergence of novel types of SE inhibitors, affecting the cis interactions of SE with their cognate genes and, thereby, repressing their expressions. Such SE inhibitors can be of particular interest in leukemia, considering the frequency of chromosomal translocations involving cis-regulatory elements, and can potentially be of interest in other types of blood disorders.

Additionally, we mentioned their existing limits, principally, that they do not target specifically oncogenic SE; therefore, not excluding the possibility of altering normal SE and enhancer regions that might be involved in essential processes of non-leukemic cell functions. An interesting step forward in the use of SE inhibitors and cancer research would be to gain specificity, by potentially finding a way to target specifically oncogenic SE, without affecting the SE found in untransformed cells. However, here again, such a challenge would require specifically identifying oncogenic SE and being able to distinguish them from normal SE.

We know that SE regulate their target genes via the formation of chromatin loops, mediated by cohesin complexes and NIPBL [44]. What if we find a way to specifically impair chromatin loops that contain oncogenic SE? We can hypothesize that targeting cohesin complexes at the base of those abnormal loops, the NIPBL proteins loading cohesins near those oncogenic SE, or CTCF insulators could be of interest. Such objectives are currently challenging, since this would require specifically inhibiting some groups of proteins localized at precise genomic locations, without affecting the same proteins only separated by several kb. Further work is, therefore, needed to allow precise targeting of these elements and the use of such strategies as therapeutics in cancer.
